# Inadvertent malposition of a permanent pacemaker ventricular lead into the left ventricle which was initially missed and diagnosed two years later: a case report

**DOI:** 10.1186/1752-1947-5-54

**Published:** 2011-02-09

**Authors:** Medhat F Zaher, Basem N Azab, Marc B Bogin, Soad G Bekheit

**Affiliations:** 1 Cardiology Department, Staten Island University Hospital, 475 Seaview Avenue, Staten Island, NY 10305, USA

## Abstract

**Introduction:**

Inadvertent malposition of a pacemaker ventricular lead into the left ventricle is an uncommon event, and its actual incidence is probably unknown. It may be underestimated and underreported because of a possible asymptomatic course. A 12-lead electrocardiogram is important to confirm proper placement.

**Case presentation:**

We report a case of a 60-year-old Caucasian man with a malpositioned transvenous permanent pacing lead into the left ventricle via a patent foramen ovale that was not suspected during implantation and went undiagnosed for two years without complications. The patient remained asymptomatic as he was being treated with oral anticoagulation therapy for atrial fibrillation. The decision was made to leave the pacing lead in place and continue lifelong warfarin therapy.

**Conclusions:**

Inadvertent insertion of pacing wires into the left ventricle is a potentially dangerous complication that may happen under fluoroscopic guidance and may be overlooked by routine pacemaker interrogation. It is advisable to obtain a 12-lead electrocardiogram during or immediately after transvenous pacemaker implantation rather than use a routine pacemaker interrogation or a limited electrocardiogram.

## Introduction

Implantation of transvenous pacing leads and implantable cardioverter-defibrillator wires is the most common surgery involving the heart [1]. It is estimated that more than 100,000 implantable cardioverter-defibrillator and more than 200,000 permanent cardiac pacemaker implantations are performed in the USA annually [2]. This procedure is performed by cardiologists, cardiothoracic surgeons, intensivists and general surgeons worldwide. The electrocardiogram (ECG) pattern of right ventricle (RV) pacing should show left bundle branch block (LBBB) and that of left ventricle (LV) pacing should show right bundle branch block (RBBB). The RBBB pattern after RV pacing could be secondary to inadvertent LV pacing or much more commonly with true RV pacing. Malposition of a ventricular lead into the LV is an uncommon event, and its actual incidence is probably unknown. It may be underestimated because of underreporting. Inadvertent LV pacing can result from unintentional placement of the ventricular lead into the LV through a patent foramen ovale or from atrial septal defects, or after perforating the interatrial septum, especially at the fossa ovalis [3]. This may especially occur in patients with dilated hearts, which may make fluoroscopic examination difficult and misleading. In these conditions, the lead passes through the atrial septum to the left atrium, then to the LV through the mitral valve. LV pacing after permanent transvenous pacemaker implantation has also been reported after ventricular septum or RV free wall perforation by the lead with subsequent LV pacing [4,5]. Moreover, unintentional placement of the ventricular lead into the distal coronary sinus or other cardiac veins has also been reported and may present with an ECG pattern of RBBB in paced mode [6]. Misplacement of the lead via the subclavian artery through the aortic valve into the LV may also result in LV pacing and a subsequent RBBB pattern shown on an ECG in pace mode [7].

The RBBB pattern during RV pacing has been correctly differentiated from LV pacing by Okmen *et al*. [8] using the following criteria: left superior axis deviation in the frontal plane between -30 and -90 degrees, precordial transition at V3, the absence of S wave in lead I and qR or RS in V1 (sensitivities and specificities are 97, 100%; 97, 100%; 94, 100%; and 97, 100%, respectively).

There are several electrophysiologic theories that explain the occurrence of an RBBB pattern during RV pacing. One explanation suggests that the stimulation impulse may travel into the right bundle branch, migrate retrogradely to the atrioventricular node and then downward antegradely into the left bundle [9]. Another theory states that some portions of the anatomical left septum extend into the right ventricular endocardium. Stimulating these septal areas can be expected to show QRS patterns similar to those observed after initial LV stimulation [10]. Similarly, the occurrence of this pattern can result from preferential activation of the left bundle branch through excitation of some of its ramifications that extend to the right side of the ventricular septum, especially if the right bundle is diseased [11].

**Figure 1:**
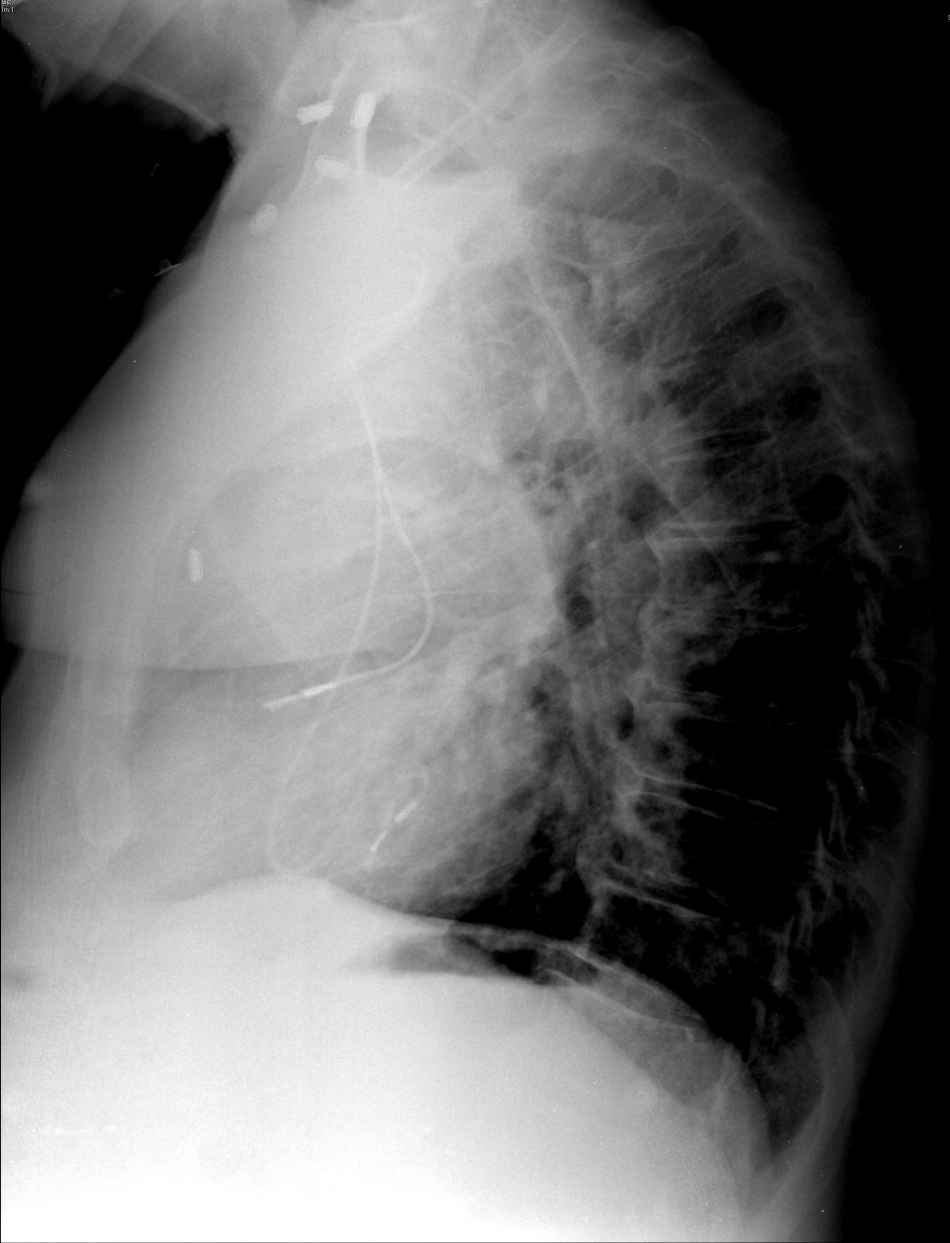
**Chest radiograph lateral projection showing the ventricular lead to be pointing posteriorly, suggesting a left ventricular site**.

The diagnosis of an inadvertently misplaced lead in the LV is simple but requires a high index of suspicion. Chest radiographs with posteroanterior and posterolateral projections should help differentiate RV from LV lead position. In our case, the tip of the ventricular lead was directed posteriorly after looping in the right atrium, which should have raised suspicion of malposition into the LV (Figure 1). The diagnosis of malpositioned pacing leads can easily be missed during routine pacemaker interrogation because of the use of modified or a limited number of surface leads. A 12-lead ECG in ventricular pacing mode that shows an RBBB pattern should raise suspicion about the ventricular lead position. Consequently, echocardiography or other imaging modalities will confirm the exact position of the wire.

Although unusual, serious complications may develop secondary to lead misplacement into the LV. These complications include systemic thromboembolism, perforation of the mitral valve leaflets, mitral insufficiency, aortic valve endocarditis, diaphragmatic pacing and loss of capture [3,12]. The exact risk of thromboembolism secondary to the presence of a pacing lead in the LV is unknown, but the incidence may reach up to 37% as suggested by previous reports [12]. On the other hand, there have been several reports in the literature about inadvertently placed pacemakers and implantable cardioverter-defibrillator leads in the LV that were accidentally discovered after up to 17 years without systemic thromboembolic events in the absence of anticoagulation therapy [13].

## Case presentation

A 60-year-old Caucasian man was admitted to hospital for new-onset of atrial fibrillation. Normal sinus rhythm was achieved after treatment with amiodarone and diltiazem. Transthoracic echocardiography showed a LV ejection fraction of 35%-40% with no valvular disease. Coronary angiography revealed nonobstructive coronary artery disease. While the patient was undergoing telemetry, he developed a three-second sinus pause and several episodes of persistent sinus bradycardia with a heart rate of 20-30 beats/min even after amiodarone and diltiazem were discontinued. The diagnosis of tachycardia-bradycardia syndrome was made, and his cardiologist decided to implant a permanent dual chamber rate adaptive pacemaker (DDDR). Under fluoroscopy, an endocardial bipolar pacing lead (model number 5594; Medtronicn (Minneapolis, Minnesota, USA) was placed into the right atrial appendage and another bipolar lead (model number 5092; Medtronic) was placed into what appeared in the operating room to be the right ventricle (RV) apex. Chest radiographs and posteroanterior and posterolateral projections after the procedure were reported to be satisfactory positioning of the pacing lead into the RV (Figure 1). On the first postoperative day, routine interrogation of the pacemaker showed loss of capture of the "RV lead." Macrodisplacement of the RV lead was suspected, and subsequently it was repositioned in the operating room with achievement of adequate capture. Stimulation threshold of the RV lead was 0.5 V at 0.06 ms. No chest X-ray was performed after the RV lead revision. The pacemaker was programmed to DDDR mode with a lower rate of 60 beats/min. A 12-lead ECG before the patient was discharged showed atrial pacing without ventricular pacing (A pace-V sense) because of programmed, managed ventricular pacing (AAI ↔ DDD) at a heart rate of 60 beats/min. The patient was discharged to home and was prescribed warfarin therapy.

During the following four months, the patient developed recurrent episodes of right isthmus-dependent atrial flutter which was successfully ablated with conversion to sinus rhythm. The electrophysiologist reported the presence of a large patent foramen ovale during the procedure.

**Figure 2:**
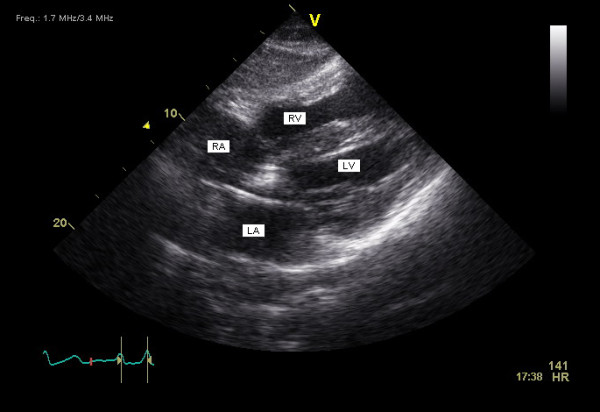
**Transthoracic echocardiography, subcostal long axis view showing the pacing lead to pass from the right atrium via the patent foramen ovale to the left atrium, then via the mitral valve to the left ventricle**. RA, right atrium; LA, left atrium; RV, right ventricle; LV, left ventricle.

**Figure 3:**
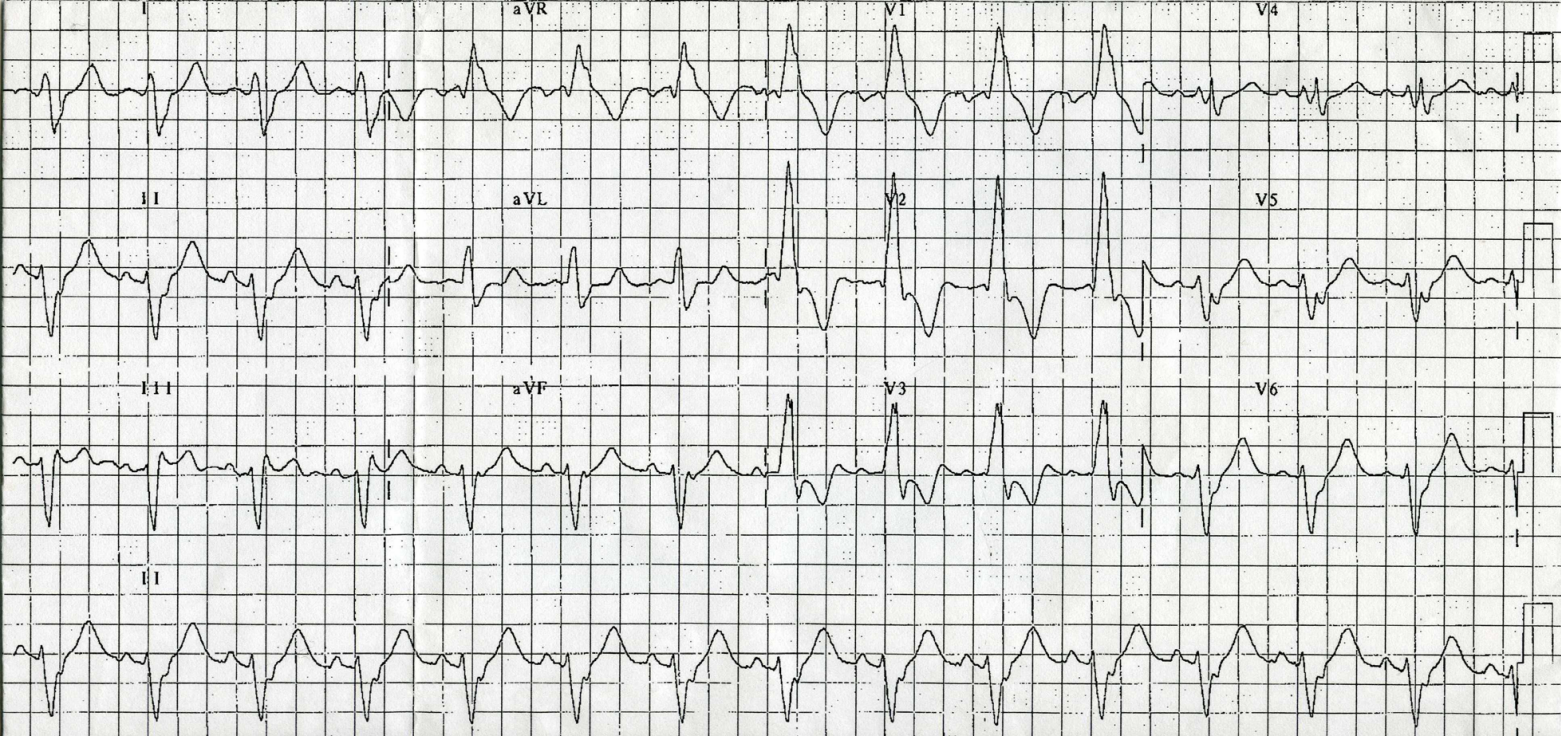
**A 12-lead electrocardiogram during magnet application**.

The patient had uneventful follow-up for two years. However, a routine follow-up echocardiogram showed the ventricular pacing wire to pass from the right atrium to the left atrium and then through the mitral valve to the LV with no visible attached thrombi (Figure 2). A 12-lead ECG during magnet application (DOO mode) showed atrioventricular pacing with RBBB morphology (Figure 3). No history of systemic embolization or transient ischemic attacks was reported. The decision was made to leave the pacing wire in place and continue lifelong warfarin therapy. To date, 40 months after insertion of the pacemaker, the patient remains asymptomatic with no manifestations suggestive of systemic embolization.

## Discussion

Although chest radiographs should help differentiate RV from LV lead position, in our case, the tip of the ventricular lead was directed posteriorly after looping in the right atrium, which should have raised suspicion of malposition into the LV (Figure 1). Also, the diagnosis of malpositioned pacing leads can easily be missed during routine pacemaker interrogation because of the use of modified or a limited number of surface leads. A 12-lead ECG in ventricular pacing mode that shows an RBBB pattern should raise suspicion about the ventricular lead position. Consequently, echocardiography or other imaging modalities will confirm the exact position of the wire. In our case, the chest radiograph was misinterpreted, and the ECG was not done in ventricular pace mode.

The therapeutic options for a misplaced lead in the LV are limited. If misplacement is diagnosed early after implantation, lead removal or adjustment is usually feasible. Adequate lifelong anticoagulation with warfarin is the therapeutic option of choice if the lead has been placed for a long time. Lead extraction should be reserved for failure of anticoagulation or during other concomitant cardiac surgery [14]. In our patient, it was decided to leave the lead in place and to continue lifelong anticoagulation.

## Conclusions

Inadvertent insertion of pacing and internal cardioverter defibrillator wires into the LV is a potentially dangerous complication that may happen even in the most experienced hands. Fluoroscopy during implantation could be difficult and misleading in localizing the site of the ventricular leads. Pacemaker interrogation after implantation does not help differentiate between RV and LV pacing. Pacing thresholds are usually normal at the time of implantation and behave normally at follow-up. It is advisable that every patient receive a 12-lead ECG in ventricular pace mode during or immediately after implantation. In case of an RBBB pattern, echocardiography should be performed for accurate localization of the ventricular lead.

## Abbreviations

LV: left ventricle; LBBB: left bundle branch block; RBBB: right bundle branch block; RV: right ventricle.

## Competing interests

The authors declare that they have no competing interests.

## Consent

Written, informed consent was obtained from the patient for publication of this case report and accompanying images. A copy of the written consent is available for review by the Editor-in-Chief of this journal.

## Authors' contributions

MZ and BA contributed by reviewing the literature and drafting the manuscript. MB and SB reviewed the manuscript and supervised the conception and design of the article. All authors read and approved the final manuscript.
